# Tabes and Syphilis

**Published:** 1882-03

**Authors:** 


### TABES AND SYPHILIS.

Prof. W Erb, *Centralbl. f. d. Med. Wissensch.*, Nos. 11 and 12, has made a recent careful study of over one hundred well-marked male cases of locomotor ataxia, and finds the result to still further confirm his previously experienced views (*Deutsche Arch. f. Klin. Medicin*, Bd. 24, 1879) as to the connection of this disease and syphilis. In the first one hundred cases he found only twelve without a previous history of syphilis or chancre; of the remaining eighty-eight, fifty-nine had had the secondary manifestations of the disease, and twenty-nine had had simply chancres. Of these last eleven had been treated constitutionally with mercury and iodide of potash, so that it is presumed that their sores were of the infecting variety; in fifteen of the others particulars as to the nature of the sore are wanting; in only three was it specified as a "soft" chancre. As regards the time of the first manifestation of tabetic symptoms after the syphilitic infection, the following are the facts: The symptoms of tabes developed between the

| | | | |
|---|---|---|---|
| 1st and 5th | year in | 17 | cases |
| 6th " 10th | " | 37 | " |
| 11th " 15th | " | 21 | " |
| 16th " 20th | " | 3 | " |
| 21st " 25th | " | 5 | " |
| After the 31st | " | 2 | " |
| Unknown | " | 3 | " |
| | | 88 | " |

In order to meet the objection that syphilis occurred so frequently in the class of people under his observation that it might be considered as an accident always to be looked for, Prof. Erb gives a comparative statement of a similar examination to that of his tabetic patients, of four hundred of his adult male patients suffering from other affections, chiefly nervous, and finds that seventy-seven per cent. of these had no history of syphilis or chancre whatever, that twelve per cent. had had secondary syphilis, and eleven per cent. simply chancre. Thus in the general adult male invalid population under his observation, the tabetic cases excluded, only twenty-three per cent. were in any way syphilitic, while in the tabetics alone eighty-eight per cent. had a history of syphilis. "In fact," he says, "if oue will not refuse all assi tance from statistics and logic in the solution of this question, it must be admitted that these figures speak most emphatically in favor of the view that there is an etiological relation between syphilis and locomotor ataxia." Of course they are not absolutely conclusive, but they go far to support the author's views. It is well worth while for others who have large opportunities for observation in this line to make similar examinations. It cannot be said that if syyphilis be proven to be at the bottom of most cases of this disease, that its prognosis is necessarily improved, but it does not render it any more unfavorable, and it will be a very interesting practical point.

